# Micronutrient Status and Other Correlates of Hemoglobin among Children with Stunting: A Cross-Sectional Study in Uganda

**DOI:** 10.3390/nu15173785

**Published:** 2023-08-30

**Authors:** Rolland Mutumba, Joseph Mbabazi, Hannah Pesu, Eva Greibe, Mette F. Olsen, André Briend, Christian Mølgaard, Christian Ritz, Ezekiel Mupere, Suzanne Filteau, Henrik Friis, Benedikte Grenov

**Affiliations:** 1 Department of Nutrition, Exercise and Sports, University of Copenhagen, 1958 Frederiksberg C, Denmark; mjosef2000@gmail.com (J.M.); hannahpesu@gmail.com (H.P.); mette.frahm.olsen@regionh.dk (M.F.O.); andre.briend@gmail.com (A.B.); cm@nexs.ku.dk (C.M.); hfr@nexs.ku.dk (H.F.); bgr@nexs.ku.dk (B.G.); 2 Department of Paediatrics and Child Health, School of Medicine, College of Health Sciences, Makerere University, Kampala P.O. Box 7072, Uganda; mupez@yahoo.com; 3 Department of Clinical Biochemistry, Aarhus University Hospital, 8200 Aarhus N, Denmark; evagreibe@gmail.com; 4 Tampere Center for Child, Adolescent and Maternal Health Research, Faculty of Medicine and Health Technology, Tampere University, 33100 Tampere, Finland; 5 National Institute of Public Health, University of Southern Denmark, 5230 Odense M, Denmark; ritz@sdu.dk; 6 Faculty of Epidemiology and Population Health, London School of Hygiene and Tropical Medicine, London WC1E 7HT, UK; suzanne.filteau@lshtm.ac.uk

**Keywords:** hemoglobin, anemia, stunting, micronutrients, inflammation, malaria

## Abstract

In low-income countries, undernutrition and infections play a major role in childhood anemia. Stunted children may be at particular risk of anemia. In a cross-sectional study nested in a nutrition trial among 12–59-month-old stunted children in eastern Uganda, we measured hemoglobin (Hb) and markers of iron, cobalamin, folate and vitamin A status. We assessed low micronutrient status, socio-demography, stunting severity, inflammation and malaria as correlates of Hb and anemia using linear and logistic regression analyses, respectively. Of 750 stunted children, the mean ± SD age was 32.0 ± 11.7 months and 55% (n = 412) were male. The mean Hb was 104 ± 15 g/L and 65% had anemia, Hb < 110 g/L. In a multivariable model with age, sex and inflammation, the following were associated with lower Hb: serum ferritin < 12 µg/L (−5.6 g/L, 95% CI: −8.6; −2.6), transferrin receptors > 8.3 mg/L (−6.2 g/L, 95% CI: −8.4; −4.0), plasma folate <20 nmol/L (−4.6 g/L, 95% CI: −8.1;−1.1), cobalamin < 222 pmol/L (−3.0 g/L, 95% CI: −5.4; −0.7) and serum retinol-binding protein < 0.7 µmol/L (−2.0 g/L, 95% CI: −4.1; 0.2). In addition, severe stunting, inflammation and malaria were negative correlates. Anemia is common among stunted children in eastern Uganda; micronutrient deficiencies, inflammation and malaria are associated with low Hb.

## 1. Introduction

Undernutrition and anemia are prevalent in low- and middle-income countries and contribute to under-five morbidity and mortality [1,2,3,4]. Globally, approximately 149 million children under five years are affected by stunting [5], whilst 40% have anemia [6]. Studies in low-income settings show that children with stunting have higher odds of developing anemia [7,8,9]. Like stunting, anemia is negatively associated with children’s physical and cognitive development [10]. Poor-quality diets lacking essential nutrients and infections contribute to both stunting and childhood anemia; hence, these are likely to co-exist in the same child [4,11]. Infections result in inflammation which downregulates the growth factor insulin-like growth factor-1, but also reduces intake and absorption and the increased excretion of nutrients of importance for growth [12]. Multiple interventions are suggested to control stunting that include ensuring good maternal nutrition; exclusive breastfeeding and good complementary feeding practices; and providing sufficient quality foods for growth and water, sanitation and hygiene interventions to prevent and protect against infections [13]. However, there are currently no programs or recommendations for treating stunted children.

We recently showed that a large proportion of children with stunting had deficiencies of iron, vitamin A or cobalamin [14]. Iron is a key component of hem in red blood cells and iron deficiency is a prominent cause of childhood anemia [15]. Cobalamin and folate are required for erythropoiesis and deficiency inhibits purine and thymidylate synthesis, impairs DNA synthesis and causes erythroblast apoptosis resulting in megaloblastic anemia [16]. Vitamin A enhances growth and the differentiation of erythrocyte progenitor cells, plays a role in iron metabolism and improves immunity [17,18]. Malaria [2,19], intestinal parasites [20,21], inflammation [22] and hemoglobinopathies [23] are also important contributors to childhood anemia. Among the nutritional causes of anemia, few studies try to account for the contribution of a wide range of micronutrients. About half of all anemia is considered due to iron deficiency [15,24]. However, based on pooled data from 16 surveys, the proportion of anemia due to iron deficiency varies with several factors including overall anemia prevalence and the prevalence of infections; hence, the proportion may vary in different settings and sub-groups [25]. To generate targeted interventions that reduce childhood anemia among stunted children, it is important to establish the contribution of key micronutrients on hemoglobin (Hb) and also assess for other hemoglobin (Hb) correlates.

We aimed to assess micronutrient status and other correlates of Hb among children aged 12–59 months with stunting.

## 2. Materials and Methods

### 2.1. Study Design and Ethics

This was a cross-sectional study using baseline data from a nutritional intervention trial among already stunted children (ISRCTN13093195). The trial recruited 750 children with stunting and was approved by the School of Medicine Research and Ethics Committee of Makerere University (REC REF 2019-013) and the Uganda National Council of Science and Technology (SS 4927) [26]. The Danish National Committee on Biomedical Research Ethics (1906848) gave consultative approval. Caregivers gave written informed consent before enrolling their children into the study. Verbal and written information was given in Lusoga, Luganda or English. The trial was conducted in accordance with the principles of the Helsinki declaration and local guidelines for conducting human research. Data were collected from February to September 2020.

### 2.2. Study Setting

The study was conducted in Jinja District, eastern central Uganda. The prevalence of stunting in eastern central Uganda is estimated at 29% [27]. Two community health facilities, Buwenge and Walukuba health centers, were used as study sites. Children were recruited from the surrounding communities within the health facility catchment area. The two health center catchment areas had both rural and urban areas.

### 2.3. Study Participants and Data Collection

Children were enrolled into the study if 12- to 59-months old, stunted (length/height-for-age (HAZ) z-scores < −2, World Health Organization, WHO) and caregiver provided informed consent. We excluded children who were identified as having severe acute malnutrition (weight-for-length/height z-score, WHZ, < −3, mid-upper arm circumference, MUAC, <115 mm, or bipedal pitting oedema), had a disability impending length/height measurement or had a medical complication requiring hospitalization. In addition, children participating in another study, with history of allergy to peanuts or milk or whose family planned to leave the catchment area within six months were excluded. Only one child was included from each household. Village Health Teams mobilized communities for initial screening within the villages. Children found to have stunting based on HAZ were referred to study sites for final eligibility assessment. Length/height was measured in triplicate by trained staff to the nearest 0.1 cm using an infant/child ShorrBoard (Weigh and Measure, Olney, MD, USA). Weight was measured to the nearest 100 g using an electronic scale (SECA 876; Hamburg, Germany) and MUAC was measured to the nearest 0.1 cm using a standard measuring tape (UNICEF SD, Copenhagen, Denmark) on the left arm. Sociodemographic data were collected by trained staff using interviewer-administered questionnaires. Following a home visit, towns with a population over 2000 persons were considered as urban areas. At inclusion, blood was collected from the forearm and divided into plain serum tubes, lithium-heparin tubes and ethylenediaminetetraacetic acid (EDTA) tubes. A maximum of 6.0 mL was collected from each child. Blood was processed on the same day, usually within 3–4 h, into either serum or plasma by centrifugation at 3500 rpm for 10 min, aliquoted into cryovials and temporarily stored at −20 °C. Using cold boxes, processed samples were transferred weekly to IBRH3AU biorepository at Makerere University in Kampala for storage at −80 °C. At the end of the study, processed samples were transferred to Denmark and Germany on dry ice for micronutrient biomarkers and acute phase proteins analysis.

### 2.4. Hb Measurement

Hb as the outcome variable was estimated from EDTA tube whole blood using a HemoCue (Hb201+, Ängelholm, Sweden) device. The HemoCue device was calibrated with a control solution on a weekly basis. Hb below 110 g/L was considered as anemia [28].

### 2.5. Malaria, Inflammation, and Biomarkers of Micronutrient Status Measurements

Malaria was diagnosed from EDTA whole blood using a rapid diagnostic test (SD BIOLINE MALARIA AG PF, Abbott, Lake Forest, IL, USA). Serum C-reactive protein (S-CRP), serum α-1 acid glycoprotein (S-AGP), serum ferritin (S-FE), serum soluble transferrin receptor (S-TfR) and serum retinol-binding protein (S-RBP) were determined at the VitMin Lab in Willstaedt, Germany, using a combined sandwich enzyme-linked immunosorbent assay [29]. Inter- and intra-assay coefficients of variation were 5–14%. Inflammation was defined by levels of CRP and AGP using the following categories: S-CRP < 2, 2–<5, 5–<10, 10–15 and >15 mg/L; S-AGP < 0.8 0.8–1.2 and >1.2 g/L. S-FE < 12 µg/L [28] and S-TfR > 8.3 mg/L [29] were used as cut-offs for depleted iron stores and tissue iron deficiency, respectively. S-RBP < 0.7 µmol/L was used to define low vitamin A status [28]. Inflammation corrected-S-FE and -S-RBP were generated following a linear regression model described by Cichon et al. [30] and cut-offs of S-CRP > 5 mg/L and S-AGP > 1 g/L were used to define inflammation. Plasma cobalamin (P-Cob), plasma methylmalonic acid (P-MMA) and plasma folate (P-Fol) levels were measured at the Department of Clinical Biochemistry, Aarhus University Hospital, Denmark, employing the Advia Centaur CP Immunoassay System (Siemens) (P-Cob and P-Fol) and Liquid Chromatography–Tandem Mass Spectrometry on the AB SCIEX Triple Quad 5500 System (AB SCIEX) (P-MMA). The total imprecisions were 7.5% and 11.8%. Cut-offs of low and marginal P-Cob < 222 pmol/L [31] and high P-MMA > 0.75 µmol/L [32] were used to define low cobalamin status. Low folate was considered as P-Fol < 20 nmol/L.

### 2.6. Statistical Analysis

Data were double entered using Epidata (Epidata Association, Odense, Denmark). All statistical analyses were carried out using STATA v15.1 (StataCorp, College Station, TX, USA). Background characteristics were summarized as mean (SD, standard deviation), median [IQR, interquartile range] for continuous variables and frequency % (n) for categorical variables. Age, sex, stunting severity, breastfeeding, malaria, inflammation and biomarkers of micronutrient status were assessed as potential correlates of Hb. Multiple linear regression analysis was used to determine correlates of Hb; we reported the β coefficient and 95% confidence interval (CI) for each correlate. For each analysis, we used three models: model 1 adjusted for age and sex; model 2 additionally adjusted for inflammation (S-CRP and S-AGP); model 3 adjusted for age, sex, inflammation and markers of low iron, cobalamin, folate and vitamin A status. Similar analyses for the categorical outcome, anemia, were conducted using logistic regression and adjusted odds ratios (AOR) reported. Missing data were not imputed and there was no adjustment for multiplicity. Statistical significance was set at *p* < 0.05.

## 3. Results

Of the 750 children with stunting, the mean (±SD) age was 32.0 ± 11.7 months and 55% (*n* = 412) were male (Table 1). Forty-two percent (*n* = 314) of children had severe stunting (HAZ < −3), 45% (*n* = 335) were from a rural setting and 55% (*n* = 413) were from the communities within the Buwenge health center catchment area. Data on Hb were available for 99% (*n* = 743) of the children. The mean Hb was 104 ± 15 g/L and 65% (*n* = 479) had anemia.

Younger children (12–23 months) compared to older children (36–59 months) had 4.8 (95% CI: 2.4; 7.3) g/L lower Hb after adjusting for sex and inflammation (Table 2). Likewise, male sex was associated with 2.4 (95% CI: 0.5; 4.4) g/L lower Hb after adjusting for age and inflammation. With adjustments for age and sex only, children residing in a rural setting had 5.4 (95% CI: 3.3, 7.5) g/L lower Hb, but this halved (−2.4 g/L, 95% CI: −4.5, 0.3) after further adjusting for inflammation. Compared to moderately stunted children, severely stunted children had 2.7 (95% CI: 0.6, 4.7) g/L lower Hb. A positive malaria test was also associated with 7.9 (95% CI: 5.8, 10) g/L lower Hb, of which approximately half was attributed to concurrent inflammation. Similarly, in the categorical analysis, a positive malaria test was associated with 70% higher odds of anemia (AOR 1.70 (95% CI: 1.17; 2.47)) (Supplementary Table S1).

After adjusting for age, sex and inflammation, S-FE < 12 µg/L was associated with 8.9 (95% CI: 6.0, 12) g/L lower Hb and S-TfR > 8.3 mg/L was associated with 7.3 (95% CI: 5.2, 9.4) g/L lower Hb (Table 3). However, in the full model with mutual adjustment for the two iron markers as well as adjustment for markers of cobalamin, folate and vitamin A, the estimates of association were somewhat lower. In the same model, P-Cob < 222 pmol/L was associated with 3.0 (95% CI: 0.7; 5.4) g/L lower Hb, whereas elevated plasma MMA was not a correlate. Plasma folate categories < 20 and 20–30 nmol/L were associated with 2.8 (95% CI: 0.6, 5.0) g/L and 4.6 (95% CI: 1.1, 8.1) g/L lower Hb, respectively, in the fully adjusted model. Likewise, S-RBP was associated with lower Hb, though marginally significant (−2.0 g/L (95% CI: −4.1; 0.2)). The intercept of the full model, which reflects mean Hb among 36–59-month-old girls without inflammation and any micronutrient deficiencies, was 117.4 (95% CI: 114.6; 120.3) g/L, compared to 107.6 (95% CI: 105.6; 109.6) g/L in a model with only age and sex. Hence, adding both markers of inflammation and all markers of micronutrients deficiencies to the model with only age and sex increased the intercept approximately 9.8 g/L (*p* < 0.001), corresponding to two-thirds of a standard deviation. Additionally, there was a 69% reduction in risk of anemia (AOR 0.31, 95% CI: 0.19; 0.51) (Supplementary Table S2).

## 4. Discussion

We found that two thirds of the children with stunting had anemia. Male sex, younger age (12–23 months), rural residence, severe stunting, inflammation, a positive malaria test and biomarkers reflecting low iron, cobalamin, folate and vitamin A status were negatively associated with Hb.

Adequate levels of iron and vitamins are required to maintain normal hematopoietic function either directly or indirectly by affecting other hematopoietic micronutrients. Indirectly, cobalamin deficiency can impair the metabolism of folate by lowering the levels of methionine synthetase leading to functional folate deficiency, consequently causing ineffective erythropoiesis [33]. Likewise, vitamin A deficiency can impair intestinal iron absorption and mobilization [16]. While deficiencies of these micronutrients may occur in isolation, they usually exist in combination and will negatively impact red cell production and function. Our findings show that the combination of low iron, cobalamin, folate and vitamin A status controlled for inflammation was associated with a reduction in the overall mean Hb of stunted children by approximately 10 g/L. Therefore, improving the status of multiple hemopoietic micronutrients may be of benefit in preventing anemia in this population.

As expected, inflammation and malaria were associated with lower Hb and were key contributors to childhood anemia in this setting. Proinflammatory cytokines during inflammation and malaria infection induce hemolysis, impair iron homeostasis and suppress erythropoiesis resulting in anemia [34]. In addition, inflammation and malaria may reduce a child’s intake of micronutrients required for hemopoiesis due to anorexia. Moreover, extensive malaria-induced hemolysis increases the requirement for cobalamin and folate due to stimulated erythroid hyperplasia [16]. Malaria parasites may cause cobalamin deficiency by utilizing cobalamin as a co-factor for methionine synthase required for their metabolism and growth [35]. Furthermore, recently published data show that malaria may contribute to iron deficiency through a hepcidin-mediated blockade of iron absorption [36].

The severity of stunting negatively affected Hb as children with severe, compared to moderate, stunting had lower Hb and 40% higher odds of anemia. A background environment of poverty, undernutrition and infections keeps both stunting and anemia prevalent within the same population [3,7,9,37]. With continued exposure to the above environment, the children will keep on a pathway that worsens stunting in addition to increasing their risk for anemia. Potentially, any improvement in this background environment is likely to have a positive synergetic effect on both stunting and anemia.

Contrary to our findings, previous studies found no sex-related difference in Hb among young children [38,39,40], but were all in agreement with our findings of higher Hb in older compared to younger age. Unlike those studies which looked at ‘healthy’ young children in the general population, our children were stunted. Boys are more likely to be stunted compared to girls [41]. The vulnerability of boys to stunting is probably due to a complex interplay of socio-cultural and biological factors like how children of different genders are culturally treated, boys being more prone to infections and having more rapid growth during infancy resulting in greater energy needs, higher lean mass and presumably higher red cell mass, thus higher micronutrient requirements [42]. Such factors may also contribute to the lower Hb observed among the male children. Moreover, a survey conducted in Uganda found female children had lower odds of anemia compared to male children [43].

Overall, most of these children with stunting had anemia with only one third having no anemia. The prevalence was close to 63% observed in a health survey among 6–59-month-old children from eastern central Uganda where the study was conducted and stunting is prevalent [27]. As our study had no comparative group of non-stunted children, we were not able to assess if the prevalence of anemia differed between stunted and non-stunted children or if the associations with anemia differed with stunting. Due to a background environment of poor nutrition and infections that contributes to both stunting and anemia, we would expect stunted children to have higher levels of anemia compared to non-stunted children. Nonetheless, we still found a high level of anemia to classify as a WHO ‘severe’ public health problem requiring focused attention. As a strength, we recruited children from the community and had a large sample size; thus we think our findings can be generalizable to children with stunting in similar settings. In addition, we assessed multiple micronutrient markers and therefore were able to determine the status of most key haemopoietic micronutrients. However, we lacked a universally acceptable age-appropriate cut off for P-MMA to define low cobalamin status as this was not available. Nevertheless, P-Cob is an important co-factor for methylation in DNA synthesis during erythropoiesis, thus is likely to show a more direct cobalamin-specific effect on Hb compared to P-MMA, which also requires P-Cob as a co-factor but in a different mitochondrial metabolic pathway [44]. We also lacked data on red cell indices and therefore could not examine the relationship between specific micronutrient deficiencies and the presence of different morphological types of anemia.

In conclusion, two out of three stunted children have anemia and multiple micronutrient deficits along with malaria and other infections considerably contribute to anemia. As part of measures to prevent and control anemia in this population, programs should target improving the status of multiple key hemopoietic micronutrients and not just individual micronutrients such as iron or folate. Furthermore, there is a need for research to develop feasible and acceptable interventions to prevent micronutrient deficiencies that contribute to stunting and anemia in young children.

## Figures and Tables

**Table 1:** Background characteristics of 750 children aged 12 to 59 months with stunting.

Characteristics	N	Mean ± SD, Median [IQR], % (n)
Age (months)	750	32 ± 11.7
12–23		30 (222)
24–35		34 (259)
36–59		36 (269)
Sex, Male	750	55(412)
Residence, Rural	750	45 (335)
Maternal education, primary and above	737	54 (399)
Breastfeeding, yes	746	13 (95)
Received deworming medication in past 6 months, yes	750	52 (390)
Height-for-age (z-score)	750	−3.02 ± 0.74
<−3		41.9 (314)
Mid-upper arm circumference (cm)	750	14.4 ± 1.18
Malaria rapid test, positive	737	40 (292)
Serum C-reactive protein (mg/L)	741	1.57 [0.33; 8.25]
<2		53 (396)
2–<5		13 (94)
5-<10		12 (88)
10-<15		5 (35)
>15		17 (128)
Serum α_1_-acid glycoprotein (g/L)	741	1.2 [0.88; 1.61]
<0.8		19 (139)
0.8–1.2		32 (232)
>1.2		50 (370)
Inflammation-corrected serum ferritin ^1^ (µg/L)	741	13.6 [7.61; 22.9]
<12		43 (318)
Serum soluble transferrin receptor (mg/L)	741	14.6 ± 10.4
>8.3		62 (457)
Plasma cobalamin (pmol/L)	719	316 ± 133
<222		24 (169)
Plasma methylmalonic acid (µmol/L)	733	0.32 [0.20; 0.55]
>0.75		16 (116)
Plasma folate (nmol/L)	692	34.7 ± 11.2
<20		9 (62)
Inflammation-corrected serum retinol-binding protein^1^ (µmol/L)	741	0.85 [0.72; 1.0]
<0.7		21 (158)
Hemoglobin (g/L)	743	104 ±15
<110 g/L		65 (479)

SD Standard deviation, IQR Interquartile range. ^1^ Corrected for inflammation by regression method using both serum C-reactive protein and α_1_-acid glycoprotein.

**Table 2:** Age, sex and other correlates of hemoglobin (g/L) among children with stunting ^1^.

		Model 1 *		Model 2 ^§^	
	n	β (95% CI)	*p*	β (95% CI)	*p*
Age, months					
36–59	268	-		-	
24–35	257	−2.4 (−4.9; 0.1)	0.055	−2.2 (−4.6; 0.1)	0.061
12–23	218	−4.7 (−7.3; −2.1)	<0.001	−4.8 (−7.3; −2.4)	<0.001
Sex					
Female	334	-		-	
Male	409	−2.9 (−5.1; −0.8)	0.006	−2.4 (−4.4; −0.5)	0.016
Residence					
Urban	408	-		-	
Rural	335	−5.4 (−7.5; −3.3)	<0.001	−2.4 (−4.5; −0.3)	0.027
Stunting degree					
Moderate	431	-		-	
Severe	312	−3.6 (−5.7; −1.5)	0.001	−2.7 (−4.7; −0.6)	0.010
Breastfeeding					
No	646	-		-	
Yes	93	−2.5 (−6.2; 1.2)	0.18	−3.3 (−6.7; 0.2)	0.06
Malaria rapid test					
Negative	444	-		-	
Positive	292	−7.9 (−10; −5.8)	<0.001	−4.1 (−6.3; −1.8)	<0.001

^1^ Data shown as number (n), regression coefficient β (95% confidence interval) and *p*-value. * Model 1 Linear regression analysis adjusting for age and sex. Age was adjusted for sex and vice versa. ^§^ Model 2 Linear regression analysis adjusting for age, sex and inflammation. C-reactive protein (<2, 2–<5, 5–<10, 10–15, >15 mg/L) and α_1_-acid glycoprotein (<0.8, 0.8–1.2, >1.2 g/L) as categorical variables were used to adjust for inflammation.

**Table 3:** Biomarkers reflecting micronutrient status and inflammation as correlates of hemoglobin (g/L) among children with stunting ^1^.

		Model 1 *		Model 2 ^§^		Model 3 ^¶^	
	n	β (95% CI)	*p*	β (95% CI)	*p*	β (95% CI)	*p*
Serum ferritin (µg/L)							
≥12	615	-		-		-	
<12	122	−4.2 (−7.1; −1.2)	0.006	−8.9 (−12; −6.0)	<0.001	−5.6 (−8.6; −2.6)	<0.001
Serum soluble transferrin receptor (mg/L)							
≤8.3	282	-		-		-	
>8.3	455	−8.8 (−11; −6.7)	<0.001	−7.3 (−9.4; −5.2)	<0.001	−6.2 (−8.4; −4.0)	<0.001
Plasma cobalamin (pmol/L)							
≥222	547	-		-		-	
<222	169	−2.7 (−5.2; −0.2)	0.034	−1.6 (−4.0; 0.7)	0.16	−3.0 (−5.4: −0.7)	0.012
Plasma methylmalonic acid (µmol/L)	730						
≤0.75	614	-		-		-	
>0.75	116	−0.7 (−3.5; 2.2)	0.65	−0.2 (−2.9; 2.5)	0.87	0.1 (−2.7; 2.8)	0.96
Plasma folate (nmol/L)							
>30	424	-		-		-	
20–30	203	−4.4 (−6.8; −2.0)	<0.001	−3.0 (−5.3; −0.8)	0.009	−2.8 (−5.0; −0.6)	0.012
<20	62	−8.3 (−12; −4.5)	<0.001	−6.6 (−10; −3.0)	<0.001	−4.6 (−8.1; −1.1)	0.010
Serum retinol binding protein (µmol/L)							
≥0.7	397	-		-		-	
<0.7	340	−5.8 (−7.9; −3.8)	<0.001	−2.0 (−4.2; 0.1)	0.06	−2.0 (−4.1; 0.2)	0.06
Serum C-reactive protein (mg/L)							
<2	393	-	-	-		-	
2–<5	93	−4.3 (−7.4; −1.2)	0.006	−2.9 (−6.1; 0.3)	0.078	−2.6 (−5.8; 0.6)	0.106
5–<10	88	−5.6 (−8.8; −2.5)	0.001	−3.7 (−7.1; −0.4)	0.028	−4.9 (−8.2; −1.5)	0.004
10–15	35	−7.9 (−13; −3.2)	0.001	−5.9 (−11; −1.0)	0.018	−5.8 (−11; −0.9)	0.021
>15	128	−13 (−16; −10)	<0.001	−10 (−14; −7.3)	<0.001	−10 (−13; −6.6)	<0.001
Serum α_1_-acid glycoprotein (g/L)							
<0.8	138	-	-	-		-	
0.8–1.2	231	−3.2 (−6.1; −0.2)	0.034	−2.2 (−5.1; 0.7)	0.141	−1.6 (−4.4; 1.2)	0.25
>1.2	368	−9.6 (−12; −6.9)	<0.001	−4.9 (−8.0; −1.8)	0.002	−3.5 (−6.7; −1.1)	0.026

^1^ Data shown as number (n), regression coefficient β (95% confidence interval) and *p*-value. * Model 1 Linear regression analysis adjusting for age and sex. ^§^ Model 2 Linear regression analysis adjusting for age, sex and inflammation, C-reactive protein and α-1 acid glycoprotein as categorical variables above. ^¶^ Model 3 Linear regression analysis adjusting for age, sex, inflammation (C-reactive protein and α_1_-acid glycoprotein) and all micronutrient biomarkers. Intercept 117 (115; 120) g/L, *p* < 0.001 which reflects mean Hb among 36–59-month-old girls without inflammation and any micronutrient deficiencies.

## Data Availability

The Ugandan act on Data Protection and Privacy and the European act on General Data Protection Regulation do not allow for personal data to be made available to other researchers without prior written approval from relevant institutions and authorities. For further information, please contact the corresponding author.

## Supplementary Materials

The following supporting information can be downloaded at: https://www.mdpi.com/article/10.3390/nu15173785/s1, Table S1: Age, sex, and other correlates of anemia among children with stunting; Table S2: Biomarkers reflecting micronutrient status and inflammation as correlates of anemia among children with stunting.
