# Supplementary material for: Micronutrient Status and Other Correlates of Hemoglobin among Children with Stunting: A Cross-Sectional Study in Uganda
Source: Nutrients. 2023 Aug 30;15(17):3785. doi: 10.3390/nu15173785 (PMC10489905; doi:10.3390/nu15173785)
Supplement: Supplementary file 1 [file nutrients-15-03785-s001.zip › nutrients-2567892-supplementary.pdf]

Supplementary Tables

Supplementary Table S1. Age, sex, and other correlates of anemia among children with stunting[1].

		Model 1[*]		Model 2[§]	
	n	AOR (95% CI)	P	AOR (95% CI)	P
Age, months					
36-59	268	-		-	
23-35	257	1.26 (0.88; 1.80)	0.20	1.26 (0.86; 1.83)	0.23
12-23	218	1.61 (1.09; 2.35)	0.015	1.75 (1.16; 2.61)	0.007
Sex					
female	334	-		-	
male	409	1.58 (1.16; 2.14)	0.003	1.56 (1.13; 2.15)	0.006
Residence					
Urban	408	-		-	
Rural	335	2.06 (1.49; 2.85)	<0.001	1.53 (1.08; 2.16)	0.016
Stunting degree					
Moderate	431	-		-	
Severe	312	1.52 (1.11; 2.07)	0.009	1.40 (1.00; 1.96)	0.049
Breastfeeding					
No	646	-		-	
Yes	93	1.24 (0.71; 2.18)	0.44	1.43 (0.80; 2.56)	0.23
Malaria rapid test					
Negative	444	-		-	
Positive	292	2.60 (1.85; 3.64)	<0.001	1.70 (1.17; 2.47)	0.006

AOR, Adjusted odds ratio.

[1]Data shown as number (n), adjusted odds ratio (95% confidence interval), and P-value.

[*]Model 1 Logistic regression analysis adjusting for age and sex. Age was adjusted for sex and vice versa.

[§]Model 2 Logistic regression analysis adjusting for age, sex, and inflammation. C-reactive protein (<2, 2-<5, 5-<10, 10-15, >15 mg/L) and α_1- acid glycoprotein (<0.8, 0.8-1.2, >1.2 g/L) as categorical variables were used to adjust for inflammation.

Supplementary Table S2. Biomarkers reflecting micronutrient status and inflammation as correlates of anemia among children with stunting[1].

		**Model 1***		**Model 2§**		**Model 3¶**	
	n	AOR (95% CI)	P	AOR (95% CI)	P	AOR (95% CI)	P
Serum ferritin (µg/L)							
≥12	615	-		-		-	
<12	122	2.14 (1.31; 3.50)	0.002	3.60 (2.15; 6.01)	<0.001	2.56 (1.46; 4.49)	0.001
Serum soluble transferrin receptor (mg/L)							
≤8.3	282	-		-		-	
>8.3	455	2.60 (1.87; 3.61)	<0.001	2.30 (1.63; 3.25)	<0.001	1.94 (1.32; 2.84)	0.001
Plasma cobalamin (pmol/L)							
≥222	547	-		-		-	
<222	169	1.40 (0.96; 2.04)	0.083	1.29 (0.86; 1.93)	0.21	1.59 (1.02; 2.47)	0.039
Plasma methylmalonic acid (µmol/L)	730						
≤0.75	614	-		-		-	
>0.75	116	1.09 (0.71; 1.67)	0.68	1.00 (0.64; 1.56)	0.99	0.92 (0.55; 1.51)	0.73
Plasma folate (nmol/L)							
>30	424	-		-		-	
20-30	203	1.49 (1.04; 2.13)	0.031	1.29 (0.89; 1.88)	0.19	1.24 (0.83; 1.84)	0.29
<20	62	3.63 (1.73; 7.60)	0.001	3.06 (1.43; 6.53)	0.004	2.40 (1.09; 5.28)	0.029
Serum retinol binding protein (µmol/L)							
≥0.7	397	-		-		-	
<0.7	340	2.15 (1.57; 2.95)	<0.001	1.46 (1.03; 2.07)	0.032	1.48 (1.01; 2.17)	0.042
Serum C-reactive protein (mg/L)							
<2	393	-		-		-	
2-<5	93	1.39 (0.86; 2.23)	0.17	1.18 (0.72; 1.94)	0.51	1.09 (0.62; 1.90)	0.76
5-<10	88	1.35 (0.83; 2.19)	0.22	1.12 (0.66; 1.87)	0.67	1.11 (0.63; 1.97)	0.71
10-<15	35	1.89 (0.87; 4.10)	0.10	1.52 (0.68; 3.37)	0.30	1.38 (0.58; 3.31)	0.46
>15	128	7.40 (4.01; 13.6)	<0.001	5.81 (3.00; 11.3)	<0.001	5.36 (2.54; 11.3)	<0.001
Serum α_1-acid glycoprotein (g/L)							
<0.8	138	-		-		-	
0.8-1.2	231	1.79 (1.16; 2.76)	0.008	1.68 (1.08; 2.61)	0.021	1.75 (1.08; 2.83)	0.023
≥1.2	368	3.11 (2.06; 4.69)	<0.001	1.87 (1.16; 3.00)	<0.001	1.86 (1.09; 3.19)	0.023

AOR, Adjusted odds ratio.
[1]Data shown as number (n), adjusted odds ratio (95% confidence interval), and P-value.
*Model 1 Logistic regression analysis adjusting for age and sex.
§Model 2 Logistic regression analysis adjusting for age, sex, and inflammation, C-reactive protein, and α_1-acid glycoprotein as categorical variables above.
¶Model 3 Logistic regression analysis adjusting for age, sex, inflammation (C-reactive protein and α_1-acid glycoprotein), and all micronutrient biomarkers. Baseline adjusted odds: AOR 0.31 (0.19; 0.51), P<0.001, which reflects the odds of having anemia in 36-59 months-old girls without inflammation and any micronutrient deficiencies
